# Investigating the effect of eye drops based on iodine nanoparticles in the treatment of corneal ulcers in rabbit eyes

**DOI:** 10.1186/s12348-023-00367-w

**Published:** 2023-10-26

**Authors:** Mostafa Feghi, Sharif Makhmalzadeh, Nasrin Masihpour, Mansour Amin, Nader Mortazavinia

**Affiliations:** 1 Department of Ophthalmology, School of Medicine, Infectious Ophthalmologic Research Center, Ahvaz Jundishapur University of Medical Sciences, Ahvaz, Iran; 2 Nanotechnology Research Centre, Ahvaz Jundishapur University of Medical Sciences, Ahvaz, Iran; 3 Department of Microbiology, Ahvaz Jundishapur University of Medical Sciences, Ahvaz, IR Iran

**Keywords:** Corneal ulcer, Nanoparticles, Betadine, Staphylococcus bacteria

## Abstract

**Background:**

Various organisms, such as bacteria, viruses, and fungi, can cause corneal ulcers. One of the leading causes of vision loss and disability worldwide is corneal ulceration. Practical, accessible, and affordable treatment for this disease seems essential.

**Materials and methods:**

Fifteen New Zealand rabbits infected with Staphylococcus aureus (ATCC 25923) corneal ulcers were randomly divided into three groups of five for the present study. (I, II, and III). Group I was used as the control group (without treatment). The second group received an iodine solution (1.25%) without a nanoparticle structure (betadine). The third group received an iodine solution with a nanoparticle structure used as eye drops. Drops in the corneal ulcer group were used five times daily for 14 days.

Microbial counts and disease severity scores were measured on the first, second, fifth, and fourteenth days and compared between groups separately for each disease.

**Results:**

The results showed that the changes in microbial load were significant in the group that received betadine and nanoparticles. The microbial load was further reduced when using iodine nanoparticles than betadine. The betadine and nano-iodine groups significantly reduced the severity of the disease in rabbits with corneal ulcers (*p* < 0.05). The average changes in disease severity score were 4.8 ± 1.3, -2.6 ± 0.89, and -2.22 ± 1.22 in the untreated, nano iodine, and betadine groups, respectively. However, a significant increase in disease severity was observed in the untreated group (*p* = 0.001). It shows a significant difference (*p* < 0.001) between the nano iodine, betadine, and untreated groups. However, the difference in disease severity changes between nano iodine and non-nano iodine groups was insignificant.

**Conclusion:**

Nanoparticle iodine is more effective than non-nanoparticle iodine in reducing bacterial load. In reducing the severity of the disease, both types of iodine were superior to no treatment. But there was no apparent difference between the two groups treated with iodine.

## Introduction

### Corneal ulcer (CU)

Corneal ulcer (CU), known as keratitis, is cornea inflammation usually accompanied by infiltration. A corneal ulcer is divided into non-infectious and infectious types [1]. Multiple risk factors, including occupational and demographic risk factors, underlying systemic diseases such as diabetes, eye surface factors such as dry eye and entropion, and trachoma, can lead to CU [2]. Corneal ulcers' most common clinical symptoms are erythema, pain, photophobia, watery eyes, pus growth, and visual impairment.

The most common microorganisms that cause it are bacterial species such as Streptococcus pneumoniae, Staphylococcus aureus, Staphylococcus coagulase-negative, and Pseudomonas aeruginosa. Herpes simplex virus type 1 is a common pathogen. Fungal keratitis is less common than bacterial one. It is common in male farmers and developing countries [3–5]. Amoebae species such as Acanthamoeba are essential in causing corneal ulcers. Contact lenses are a significant risk factor for corneal ulcers caused by acanthamoeba [6].

## Iodine and povidone-iodine

PVP-I is a chemical compound comprising povidone polymer (polyvinylpyrrolidone) and triiodide (I3-). Free iodine, released from this complex in solution, has a broad antimicrobial activity. Iodine destroys microorganisms by iodination of lipids and cytoplasmic oxidation and membrane elements without harming mammalian cells [7]. The broad antimicrobial spectrum of PVP-I includes Gram-positive and Gram-negative bacteria, protozoa, fungi, and viruses [8].

It is used in many medical cases today. For example, it is used in ophthalmology to disinfect the eye and surgical site to prevent infections such as endophthalmitis. It is also widely used to avoid and treat infant conjunctivitis [9].

There are reports about the positive effect of iodine on dry eye, cataracts, and age-related macular degeneration, which may be due to its antioxidant property and better microcirculation. The cornea can absorb the iodine in the spray. Sclera, aqueous humor, and vitreous can absorb sufficient amounts of iodine. However, the retina absorbs a small amount of iodine [10].

## Nanoparticles and nanomedicines

Most of A typical topical drug is lost due to processes such as tear production and blinking. Due to this reduction in effect, eye drops require frequent doses and a high drug concentration, leading to poor patient compliance and increased side effects. Therefore, ophthalmic drugs can be delivered to specific target sites using nanocarriers such as nanoparticles, which have the potential to revolutionize the treatment of many eye diseases. Today, liposomes are the most common nanoparticles used for ocular drug delivery. More diverse types of nanoparticles are being developed. The most widely used materials used in nanomedicine are lipids (liposomes), proteins (albumin), cyclic oligosaccharides (cyclodextrin), engineered polymers (polymeric micelles, den,drimers, and hydrogels), and inorganic chemicals (NPs). Nanoparticles have received increasing attention due to their ability to increase the solubility of hydrophobic drugs, continuously release drugs by reducing toxicity and improving drug efficacy, and their ability to prolong drug retention time, increasing drug penetration through ocular barriers [11–14].

Nowadays, nanomedicines have become common; for example, nanomedicines to the treatment of various diseases such as multiple sclerosis, cancer, hepatitis, and schizophrenia. Liposomes, nanoparticles, nanocrystals, nanoemulsions, nano complexes, and polymer-protein conjugates are used in these drugs [15].

## Previous studies on the effect of iodine on corneal ulcers

### Studies on non-nanoparticle iodine

A pilot study was conducted in 2022 by Emilio Pedrotti et al. on the effectiveness of a 0.66% emulsion of PVP-I in treating infectious keratitis. One drop was prescribed six times a day. In case of new-onset hypopyon, impending corneal perforation, or rapid worsening of infiltration (i.e., deepening or expansion), during the period until the outcome, patients were excluded, and an experimental treatment or surgical management was performed. Clinical findings, especially changes in the size and depth of the corneal ulcer, were analyzed and collected in a slit lamp. Topical administration of 0.66% PVP-I during the time-to-outcome period was a safe strategy in patients with infectious keratitis. Also, this method saves on the use of antimicrobial agents with a wide range. This method is especially effective in eyes with Gram-positive bacterial infection [16]. A 2005 study by Ninel Z Gregori et al. aimed to compare the clinical efficacy of PVP-I versus placebo in treating corneal ulcers. This study randomly assigned patients with corneal ulcers to the PVP-I or placebo group. Wounds were cultured before and after a 10-min application of 5% PVP-I or artificial tears without preservatives. Then all patients were treated with standard antibiotic drugs. The number of colony-forming units (CFUs) before and after PVP-I or placebo was compared. It was shown that a single application of 5% PVP-I did not reduce corneal ulcer bacterial load more than the placebo alone. This Conclusion is probably due to the lack of deep penetration into the corneal stroma. Also, other factors may be involved [17].

### Studies on nanoparticle iodine

Only one study has been conducted on the effect of iodine nanoparticles on corneal ulcers. Bordin, in 2020 performed a case study to investigate the effectiveness of 0.66% povidone-iodine nanoemulsion in treating ocular ulcers. A 61-year-old male reported left eye discomfort, redness, watery eyes, and photophobia for five months. The diagnosis of conjunctivitis and an ocular ulcer was made using biomicroscopic testing techniques. Antibiotics and antiviral medications were used as a primary form of therapy, but they could not manage the symptoms. As a result, a 4-week, three-times-per-day povidone-iodine (PVI 0.66%) therapy regimen was recommended. In this case, the corneal ulcer healed with significant efficacy. It was determined that the disinfectant PVI 0.66%, which has broad-spectrum action against bacteria, fungus, viruses, and protozoa, efficiently treats ocular ulcer symptoms and cures the condition. It was also mentioned that this substance may be a helpful medicinal aid if the pathogen is unclear. However, additional research is necessary before using it to heal corneal ulcers [18].

Along with treating corneal ulcers, iodine particles have also been used to treat endophthalmitis. Povidone-iodine's effectiveness in the therapy of endophthalmitis has been investigated in some earlier animal research. For instance, Brozou et al. investigated the effects of injecting povidone-iodine into the eye to treat endophthalmitis brought on by Staphylococcus epidermis. Twenty white bunnies were used in the research mentioned above. Each animal received an injection of Staphylococcus epidermis into the right eye and underwent daily clinical examinations. (Anterior and posterior canine examination). When clinical signs started to show, 0.1 ml of PVI was administered intravitreal. PVI was administered to the first group of 10 rabbits at a concentration of 0.1% and to the second group of 10 rabbits at 0.2%. After the growth time, samples of the vitreous body and the retina were collected, and histological analysis was carried out. The first group's findings revealed no evidence of therapeutic advancement. Staphylococcus was detected in the vitreous body culture at 108 CFU/ml concentration. The animals in the second group had some mild irritation, but no staphylococcus was found in the retinal culture. Chronic inflammation was discovered through histological analysis. It was determined that intraocular infusion of 0.2% PVI might prevent Staphylococcus epidermis from inflicting bacterial endophthalmitis on rabbit eyes [19].

Various microorganisms cause corneal ulcers. In some cases, several organisms are at the same time.

The diagnostic methods of the organism are time-consuming (at least 48 h to detect the type and sensitivity of bacteria to antibiotics). Common antibiotics used to treat corneal ulcers are not widely available and are relatively expensive. Therefore, a drug with broad-spectrum and effective antimicrobial properties, available and low-cost, is needed to treat this disease. Solutions containing iodine have these properties. Therefore, we decided to investigate the effectiveness of using eye drops containing iodine nanoparticles on corneal ulcers.

## Materials and methods

### Preparation of nanoparticles

In this study, nanoparticles were made using the Diffusion Emulsification-So technique. A 1:1 mixture of benzyl alcohol and water was placed on the heater-stirrer at a temperature of 55 degrees Celsius, and It was allowed to mix well and saturate each other for 11 min. Then it was kept still for 21 min until the saturated phases were separated from each other (the organic phase saturated with water and the aqueous phase saturated with organic compound), and two phases were produced. Thus the lower phase containing benzyl alcohol saturated with water and the upper phase containing water saturated with benzyl alcohol was obtained. Compritol and oleic acid are melted together at a temperature of 55–65 degrees using indirect heat mixed, and then at the same temperature, the first part of the surfactant formula that includes lecithin or a 1:1 mixture of Span 20 and tween 80 was added to it. After mixing and becoming uniform, the desired amount of iodine (1% concentration). weight/weight) was added, and after complete mixing, the required amount of benzyl alcohol saturated with water was added to it and mixed well.

On the other hand, to prepare the aqueous phase, mix 1:1 propylene glycol and polyethylene glycol 300 in the amount of 3% of the total formula, with the second part of the surfactant-containing labrafil at a temperature of 55 degrees on bain-marie It was mixed. After mixing, the required amount of water saturated with benzyl alcohol was added and well mixed. The aqueous phase was added dropwise to the lipid phase. In contrast, the phase mentioned under the device, The high-speed homogenizer (HSH), was mixed well at 12,000 rpm for three minutes until the emulsion was formed. It was then placed at four °C for 31 min and then for 5 min. It was sonicated at room temperature. 18.75 mg of chitosan was dissolved in an aqueous solution (v/v) of 0.5% acetic acid. Then The resulting solution was diluted to a concentration of 2.5 ml/mg, and finally, its pH was adjusted with sodium hydroxide. to 5. The final solution was cooled to 4°C for the next steps. The emulsion formed in the previous step with 30 ml of 4 °C solution consisting of ml/mg 2.5 Chitosan was diluted to 60 ml and mixed and homogenized for 30 min at the same speed to form NLC particles. Several formulations were made in the form of pre-formulation, and it was observed that the considered range did not have the desired efficiency. Finally, A final formulation was obtained.

### Characteristics of nanoparticles

#### Particle size

The formulation of solid lipid nanoparticles without iodine had a particle size of 133 ± 8.5 nm, and after loading iodine, this size increased to 168 ± 7.2, which is a significant increase. (*P* = 0.028). On the other hand, Poly Dispersity Index (PDI) parameter obtained is equal to 0.24, which indicates the homogeneity of the particle size in the aqueous suspension of nanoparticles, but still the size of Particles below 200 nm—considering that properties such as stability, and membrane permeability are affected by particle size. Therefore, this feature is one of the most essential features of nanoparticles. The size of the particles below 200 nm obtained in this study has increased the Brownian motions and the resulting suspension's stability. On the other hand, considering that Lipid nanoparticles are responsible for the passage of iodine through the cornea, particle size is very influential in this feature. These particles, having a size of 100–200 nm, can penetrate the membrane; therefore, the nanoparticles prepared in this study help the penetration of iodine into the eye.

#### Particle morphology

The images prepared by the AFM method show spherical particles with high homogeneity, proving the formation of lipid nanoparticles. The particle size obtained by the AFM method confirms the particle size results obtained by the optical diffraction method. Also, the pictures taken after three months of storage show the stability of the nanoparticles and the non-integration of the particles into each other. Percentage of iodine loaded in nanoparticles One of the most critical factors is Entrapment Efficacy (EE%), which expresses the percentage of the drug It is loaded with nanoparticles. The EE% value of iodine in iodine nanoparticles is 81.3 ± 2.7%. Considering the lipophilic nature of iodine, this percentage seems reasonable.

### Iodine released test from lipid nanoparticles

The obtained results indicate that iodine was continuously released during the first ten hours, and after 4 h, about 21% was released. Therefore, lipid nanoparticles have acted as a reservoir for the iodine Act, continuously released it, and made it available to the environment. Considering that the retention time of nanoparticles on the eye's surface is also limited, the nanoparticles prepared in this study can provide about 21% of iodine to the eye during the first 4 h.

### Animal clinical trial

In the present study, 15 New Zealand rabbits weighing 2 to 2.5 kg were studied. The rabbits did not differ regarding breed, sex, age, or physical health, and they did not have any eye abnormalities or corneal ulcers. They were kept under the same conditions and received the same proper nutrition. In this study, rabbits were randomly divided into groups of 5 (I, II, and III). After infecting the eyes of rabbits with Staphylococcus aureus bacteria and inducing corneal ulcers, the selected formulation of iodine lipid nanoparticle was used for group III rabbits, and iodine solution (1.25%) without nanoparticle structure was used as eye drops for group II rabbits. Group I was considered the control group. Nanostructured lipid carriers (NLC) formulations without drugs were used for Group I. The sample number was determined using the G*Power program. The minimal number of rabbits needed for the research was determined to be 13 based on the variables, the effect size of 0.35 with a confidence factor of 95%, the test power of 80%, and a related study by Brozou et al.

In this study, Staphylococcus aureus (ATCC 25923) was used. In the antibiogram, this bacterium was resistant to penicillin and ampicillin antibiotics but was sensitive to gentamicin, tetracycline, clindamycin, and erythromycin antibiotics.

Ketamine (30 mg/kg) and xylazine hydrochloride (10 mg/kg) subcutaneous injections were part of the anesthesia procedure, and tetracaine eye drops (0.5%) were used for local anesthesia of the eyes. A mydriasis procedure was also carried out using tropicamide (1%) droplets. A 27 gauge needle with a 1 ml syringe was introduced into the corneal stroma's middle depth and halted at the border of the 2 mm optical zone to cause an ulcer. Next, 100 microorganisms of Staphylococcus aureus were infused into 0.02 ml of physiological serum. After 24 h of injection using the score of Johnson et al. and Sanders et al.(Table 1), rabbits suitable for further study regarding the ulcer were isolated. Accordingly, rabbits with a score greater than two and less than 5, and a positive culture of Staphylococcus aureus from corneal secretions and necrotic tissue, were selected.

**Table 1:** Johnson et al. and Sanders et al. scores include the following

Score	Secretion rating	Conjunctiva rating	Hypopyon rating	Corneal rating
0	no secretion	no hyperemia and edema	no empyema	no infiltrate or corneal edema
1	a small amount of secretion	slight hyperemia and edema	< 1/4 of anterior chamber	transparent cornea, slight edema, scattered punctuate infiltrates, clearly visible iris texture
2	a medium amount of secretion	Mild hyperemia and edema	1/4–1/2 of the anterior chamber	Moderate corneal opacity, infiltration area up to 1/4–1/2 of the entire cornea, still visible iris texture
3	A large amount of secretion	moderate conjunctival hyperemia and edema	1/2–3/4 of the anterior chamber	severe corneal opacity, corneal infiltrates up to 1/2–3/4 of the entire cornea, faint iris texture
4	upper and lower eyelid adhesion caused by secretion	severe conjunctival hyperemia and edema	> 3/4 of the anterior chamber	infiltration area > 3/4 of the entire cornea, gray-white opacity, invisible iris, and pupil
5				corneal perforation

The group with corneal ulcers applied drops five times daily. According to the described procedure, the rabbits' eyes were examined daily for up to 14 days using a slit light microscope and an indirect ophthalmoscope. Imaging was used to capture the alterations. To compare the effectiveness of the treatment, cultures were also taken from the corneal ulcer on the first, second, fifth, and fourteenth days.

SPSS17 software was used to analyze the data. The significance level was considered less than 0.05.

## Results

### Comparison of the mean microbial counts at different measurement times among three groups of rabbits with corneal ulcers

**Fig. 1:**
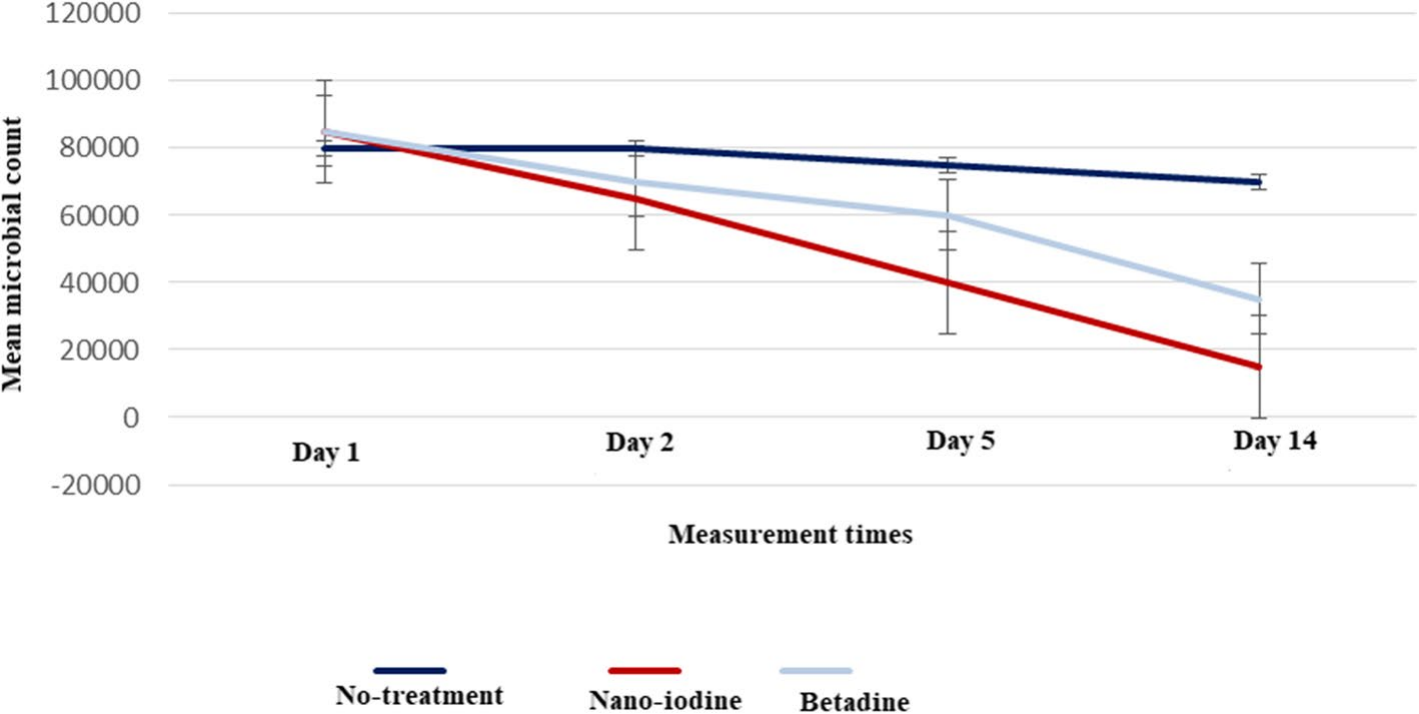
Changes in microbial load count at different measurement times in rabbits with corneal ulcers

Table 2 and Fig. 1 show the mean microbial load count of 0.1 ccs from the cornea sample at different measurement times among three groups of rabbits with corneal ulcers. The results revealed that the microbial load did not change significantly during these times in the non-treatment group (*P* = 0.09).

**Table 2:** Comparison of the mean microbial load (colony counts) of 0.1 ccs from the cornea sample at different measurement times among three groups of rabbits with corneal ulcers

Measurement time	Non-treatment	Nano-iodine	Betadine	P1	P2	P3
First day	80000±5000	85000±4000	80000±5000	0.94	0.94	1
Second day	80000±3000	65000±5000	70000±2000	0.004	0.003	0.8
Fifth day	75000±5000	40000±4000	60000±4000	0.001<	0.004	0.001<
Fourteenth day	70000±3000	15000±2000	35000±4000	0.001<	0.001<	0.001<
*P*-value^¶^	0.09	0.001<	0.001			

P1: comparison of the mean at each measurement time between the non-treatment group and the nano-iodine group, P2: the non-treatment group and the betadine group, and P3: the nano-iodine group and the non-nano-iodine group±

¶:The mean comparison between different measurement times in each group

However, there was a significant reduction in the nano-iodine and betadine groups, and the level of changes in the nano-iodine group was more significant than in the betadine group (*P* < 0.05). Comparing each of the measurement times in the nano-iodine group showed that the microbial load decreased significantly from 85.000 ± 4.000 on the first day to 65.000 ± 5.000 on the second day, 40.000 ± 4.000 on the fifth day, and 15.000 ± 2.000 on the fourteenth day (*P* < 0.001). Additionally, in the betadine group, the microbial count decreased on the second and fifth days. The fourteenth day, respectively, with a mean of 70.000 ± 2.000, 60.000 ± 4.000, and 35.000 ± 4.000, and this decrease on the second, fifth, and fourteenth days was statistically significant compared to the first day (*P* = 0.001).

Pairwise comparison of the groups in each of the measurement times showed that there was a significant difference in the reduction of the microbial load in both nano-iodine and betadine groups compared to the non-treatment group in all three times of the second, the fifth, and the fourteenth days. Also, the changes in microbial load between the two groups of nano-iodine and betadine were significant on the fifth and fourteenth days (*P* < 0.05). However, these two groups' differences were insignificant on the second day (*p* = 0.8). At the end of the study (on the 14th day), the microbial load decreased from 80.000 ± 5.000 on the first day to 70.000 ± 3.000 in the non-treatment group, from 85.000 ± 4.000 to 15.000 ± 2.000 on the fourteenth day in the nano-iodine group, and from 85.000 ± 5.000 to 35.000 ± 4.000 in the betadine group. However, the reduction in the nano-iodine group with 70.000 colony counts was higher than the other two groups, and nano-iodine significantly reduced the microbial load.

### Comparison of the mean disease severity score before and after the intervention of all three groups in rabbits with corneal ulcers

Three sets of rabbits with corneal ulcers are shown in Table 3, with the mean disease severity ratings before and after the intervention. The findings demonstrated no statistically significant difference between the three groups regarding the mean illness severity score at the start (*p* = 0.43). The disease severity score in the nano-iodine group decreased significantly, so the mean disease severity decreased from 4.4 ± 0.89 before the intervention to 1.8 ± 1.09 on the fourteenth day (*p* = 0.003). In the betadine group, the disease severity was significantly reduced compared to before, so its mean decreased from 4.2 ± 0.83 before the intervention to 2.24 ± 0.83 on the fourteenth day (*p* = 0.02). However, in the non-treatment group, a significant increase in the disease severity was observed (*p* = 0.001), so its mean increased from 4.8 ± 0.45 before the intervention to 9.6 ± 1.51 at the end of the study (fourteenth day) (*p* = 0.001). There was a significant difference between the mean changes of the disease severity score in the non-treatment, nano-iodine, and betadine groups, respectively, with the mean of 4.8 ± 1.3, -2.6 ± 0.89, and -2.22 ± 1.22 (*p* = 0.006). Pairwise comparison using post hoc test tests showed a significant difference between the non-treatment, nano-iodine, and betadine groups (*p* < 0.001). However, the disease severity changes were insignificant between the two groups of nano-iodine and betadine groups (*p* = 0.69). Table 4 shows an example of corneal changes in rabbits with corneal ulcers in three treatment groups.

**Table 3:** Comparison of the mean disease severity scores among three rabbits with corneal ulcers

Groups	Before	After	Mean changes	*P*-value^¶^
Non-treatment	4.8±0.45	9.6±1.51	4.8±1.3	0.001
Nano-iodine	4.4±0.89	1.8±1.09	-2.6±0.89	0.003
Betadine	4.2±0.83	2.24±0.83	-2.22±1.22	0.02
*P*-value^¶¶^	0.43	0.007	0.006	-

^¶^Comparison of the means in each group before and after the intervention using the Wilcoxon test

^¶¶^Comparison of means among three groups using the Kruskal–Wallis test

**Table 4:**
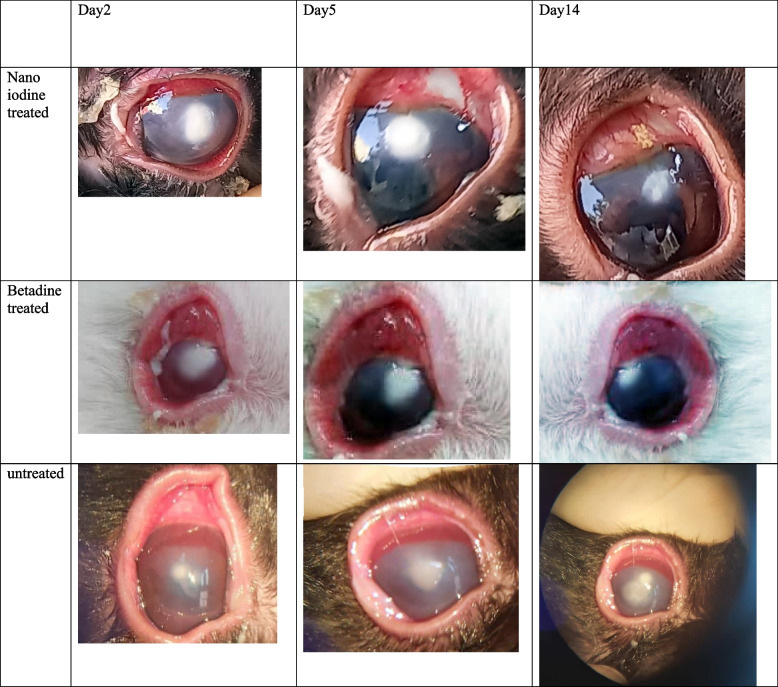
An example of corneal changes of rabbits with corneal ulcers in three treatment groups. Treated with iodine nanoparticles, treated with non-nano iodine (betadine), and untreated group

## Discussion

Corneal ulcer disease as a sight-threatening disease requires immediate treatment with the most effective treatment. Due to the increasing resistance to existing antibiotics and, on the other hand, a wide range of organisms that cause corneal ulcers requires drugs with a broad antimicrobial spectrum, cost–benefit, and availability. Iodine and its compounds, such as povidone-iodine, have been widely used as antimicrobials for many years. Therefore, by increasing the permeability and stability of iodine through methods such as nanoparticle formulation, iodine can be used to treat many diseases, including corneal ulcers. A limited number of studies have investigated the effect of iodine nanoparticles on corneal ulcer healing as a case report—a survey by Bordin et al. In 2020, it was shown that using 0.66% PVI nanoemulsion in a 61-year-old man with a corneal ulcer effectively treats and resolves the signs and symptoms of corneal ulcer [18]. Compared to the previous one, more studies have been conducted on the effect of non-nanoparticle iodine in corneal wound healing. Based on these studies, there is disagreement about the positive impact of non-nano iodine in treating corneal ulcers [16, 17]. It was shown in the present study. There is a significant difference in the reduction of microbial load between both groups receiving nano iodine and non-nano particle iodine (betadine) and untreated groups. Iodine nanoparticles caused a more significant decrease in the microbial load compared to the group treated with betadine. According to the antibiogram of Staphylococcus aureus bacteria (ATCC 25923) used in the study, it can be concluded that the effect of iodine, especially its nanoparticle type, in the treatment of corneal ulcers is comparable to antibiotics such as tetracycline, clindamycin, gentamicin, and erythromycin. The severity of the disease was significantly reduced in both the betadine and nano-iodine groups compared to the untreated group. But there was no significant difference between the nano-iodine and betadine groups in terms of reducing the severity of the disease. This issue could be due to the method of scoring the severity of the disease. Therefore It is recommended to carry out more clinical investigations with different scoring methods in terms of the severity of the disease, as well as the use of different dosages and formulations of iodine to achieve the maximum therapeutic effect.

## Conclusion

Iodine-based nanoparticles are significantly effective in reducing the severity of corneal ulcers and the microbial load of corneal ulcers in rabbits. Compared to non-nanoparticle iodine, nanoparticle iodine caused a more significant decrease in microbial load. In terms of the effect on the severity of the disease, there was no significant difference between the two.

## Data Availability

Data sets generated and analyzed during the current study are available from the corresponding author upon request.
